# Recurrent abdominal pain in children in Wuhu, China was not associated with *Helicobacter pylori* infection, but associated with <1 h/day physical activity and academic stress

**DOI:** 10.3389/fped.2024.1481125

**Published:** 2024-12-06

**Authors:** Xiaohui Bai, Huiru Cao, Liuming Zhu, Xiaomin Wu, Guixiang Wang, Wenchao Yu, Yong Gu

**Affiliations:** ^1^ Department of Pediatrics, Graduate School, Wannan Medical College, Wuhu, Anhui, China; ^2^ Laboratory of Digestion, Department of Gastroenterology, Yijishan Hospital, The First Affiliated Hospital of Wannan Medical College, Wuhu, Anhui, China; ^3^ Department of Pediatrics, Wuhu No.1 Peoples’ Hospital, Wuhu, Anhui, China; ^4^ Department of Gastroenterology, The Second Affiliated Hospital of Wannan Medical College, Wuhu, Anhui, China; ^5^ Department of Pediatrics, Yijishan Hospital, The First Affiliated Hospital of Wannan Medical College, Wuhu, Anhui, China

**Keywords:** children, recurrent abdominal pain, *Helicobacter pylori infection*, physical activity, academic stress

## Abstract

**Background:**

Recurrent abdominal pain (RAP) is one of the most common gastrointestinal disorders in children. The aim of this study was to investigate the relationship between RAP and *Helicobacter pylori* infection in children in Wuhu, China as well as the risk factors for *Helicobacter pylori* infection in this region.

**Materials and methods:**

In this cross-sectional survey, we randomly selected children aged 6–17 years who underwent health examinations at three public hospital examination centers in Wuhu city, Anhui Province, China. *Helicobacter pylori* infection was assessed by a ^13^C-urea breath test (UBT) kit. Questionnaires were custom designed to obtain data on behavioral, sociodemographic, and environmental characteristics, and to investigate the relationship between RAP and *Helicobacter pylori* infection in children.

**Results:**

A total of 1,187 children aged 6–17 years were enrolled, among these children, 182 were diagnosed with RAP, with an incidence rate of 15.3%. A total of 266 children were infected with *Helicobacter pylori*, with an infection rate of 22.4%. Multivariate regression analysis revealed that physical activity for <1 h/day and academic stress are associated with RAP in children, whereas *Helicobacter pylori* infection, age, sex, body mass index (BMI), and fast-food consumption are not associated with RAP in children. Our research also found that the risk of *Helicobacter pylori* infection increases with age in children. Risk factors for *Helicobacter pylori infection* in children include left-behind children, poor hygiene habits, family history of *Helicobacter pylori* infection, and mother with low cultural levels.

**Conclusions:**

Recurrent abdominal pain in children in Wuhu, China was not associated with *Helicobacter pylori* infection, but strongly associated with <1 h/day physical activity and academic stress.

## Introduction

Abdominal pain is a common problem in pediatric practice. Most children present with transient acute abdominal pain, but some children experience recurring symptoms of abdominal pain as recurrent abdominal pain (RAP) (1). RAP in children is defined as at least three episodes of pain that occur over at least three months and affect ability of normal activitie. RAP often causes psychological problems for children and their parents, such as nervousness and anxiety (2, 3). Effective treatment methods for RAP are currently lacking, and patients repeatedly undergo medical examinations and treatments, which not only wastes medical resources but also increases the economic burden on families and affects the quality of life of the children and their parents (4–6). RAP is also an important factor in children's absenteeism and poor academic performance (7–9).

It is generally accepted that RAP represents a group of functional gastrointestinal disorders in children that have an unclear etiology. The pathogenesis of RAP in children may be related to factors such as mental stress, abnormal brain–gut axis interactions, eosinophilic gastroenteritis, and carbohydrate intolerance (10–13). The etiology of RAP is not clear, and clarifying the risk factors for RAP will provide reliable evidence for its treatment.

Some scholars have reported that *Helicobacter pylori* infection is associated with RAP in children (14–16), but there are also different opinions, suggesting that most children infected with *Helicobacter pylori* are asymptomatic and do not experience RAP (17, 18). At present, there is still controversy regarding the relationship between RAP and *Helicobacter pylori* infection. Especially in China, where there is a lack of reports on the relationship between RAP in children and *Helicobacter pylori* infection. The aim of this study was to investigate the relationship between RAP and *Helicobacter pylori* infection in children in Wuhu, China, and to determine the risk factors and epidemiological characteristics of *Helicobacter pylori* infection in children in this region. This study will provide epidemiological evidence for the prevention and treatment of RAP and *Helicobacter pylori* infection.

## Materials and methods

### Study objects and groups

From February 1, 2024, to April 31, 2024, we randomly selected healthy children aged 6–17 years from the physical examination centers of three public hospitals in Wuhu (The First Affiliated Hospital of Wannan Medical College, the Second Affiliated Hospital of Wannan Medical College, and the First People's Hospital of Wuhu city) as the research subjects. A total of 1,187 children were included, and all enrolled children underwent a ^13^C-urea breath test (UBT).

The exclusion criteria were as follows: (1) having a guardian who was unwilling to sign the informed consent form; (2) having organic lesions, such as peptic ulcers, inflammatory bowel disease, liver or gallbladder disease, respiratory system disease, cardiovascular disease, or urinary system disease; (3) having taken antibiotics in the past month or proton pump inhibitors, H2 receptor antagonists and drugs containing bismuth orally in the past two weeks; (4) having a history of *Helicobacter pylori* eradication.

**Figure 1:**
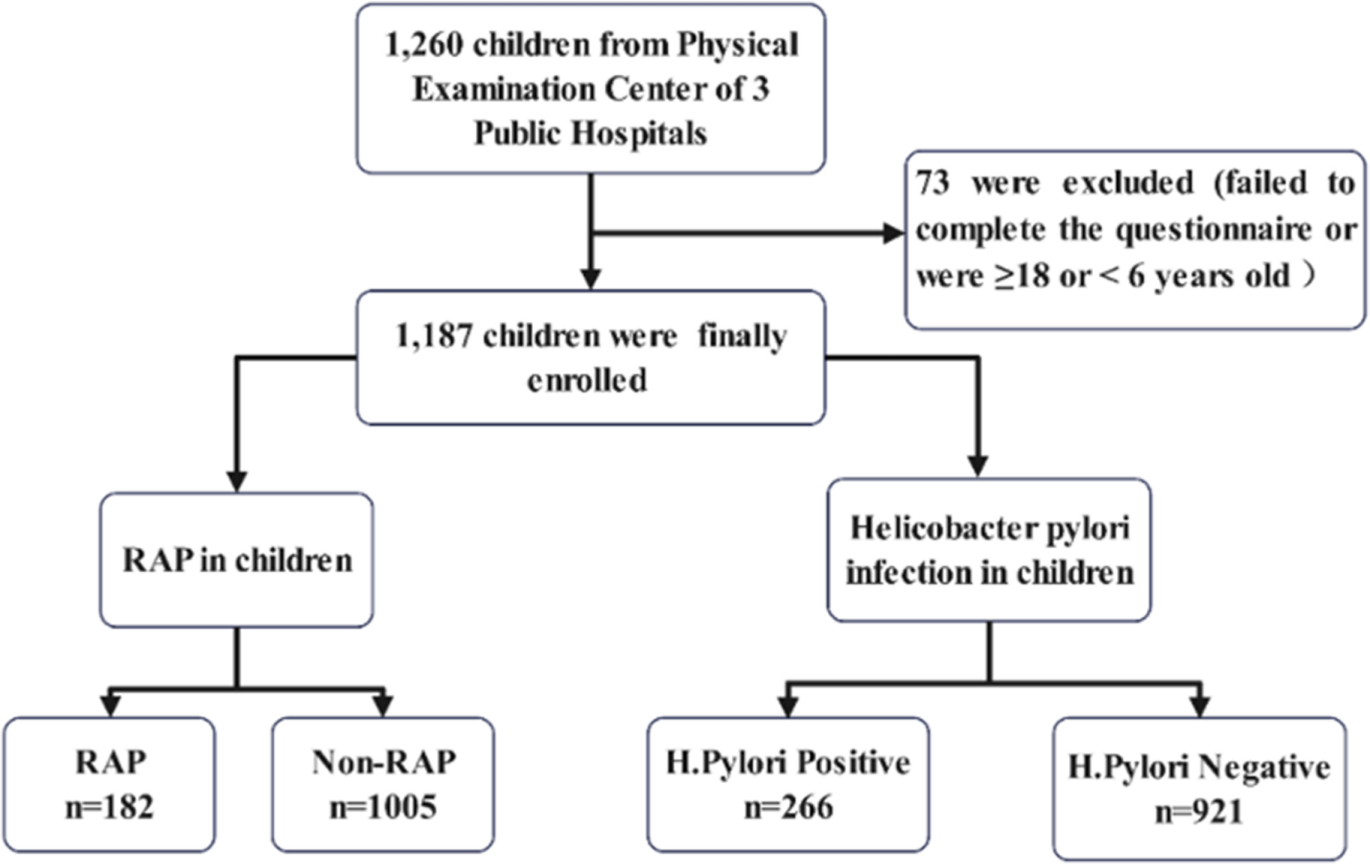
Flowchart of participant recruitment and group assignment.

According to whether children have RAP, all the children were divided into a RAP group and a non-RAP group to investigate the risk factors for RAP. According to the results of ^13^C-UBT, all the children were divided into a *Helicobacter pylori*-positive group and a *Helicobacter pylori*-negative group to investigate the risk factors for *Helicobacter pylori* infection (Figure 1).

### Diagnosis of RAP and *Helicobacter pylori* infection

The diagnostic criteria for RAP were as follows: (1) having at least 3 episodes within 3 months; (2) experiencing nonspecific, intermittent pain attacks rarely accompanied by colic; (3) having pain mostly located in the umbilical area or around the navel without a fixed area, with abdominal examination revealing mild tenderness or no abnormalities in the umbilical area; and (4) experiencing nausea, vomiting, and bloating as accompanying symptoms (1).

The diagnostic methods and criteria for *Helicobacter pylori* infection were as follows: children were tested for *Helicobacter pylori* infection by ^13^C-UBT kit. Children with a delta over the baseline (DOB) ≥4.0 were considered *Helicobacter pylori*-positive, and those with a DOB <4.0 were considered *Helicobacter pylori*-negative.

### Data collection

A relevant questionnaire was developed according to the research content. Face-to-face interviews were conducted with all the participating children, and the questionnaires were completed with the consent and assistance of their guardians. All interviews were conducted by trained investigators. The questionnaire collected data on the following: name, sex, age, height, weight, abdominal pain characteristics (location, onset time and frequency, accompanying symptoms), ^13^C-UBT results, physical activity level (≥1 h/day, <1 h/day), academic stress status (1 = not at all; 2 = a little; 3 = some; and 4 = a lot; a score of 3–4 was considered to indicate academic stress), fast-food consumption (fast food is defined as pre made meals that can be quickly served to customers, such as hamburgers, pizzas, boxed meals, and instant noodles. The frequency of consuming fast food is divided into three situations. occasionally = ≤2 times/week, sometimes = 3–4 times/week, often ≥5 times/week), place of residence (rural or urban), left-behind status, personal hygiene habits (frequency of tooth brushing and washing hands before meals), parental education levels, parental history of *Helicobacter pylori* infection, dishwashing methods (washing dishes with a basin or running water), tableware disinfection status, and total household income level.

The study was approved by the Ethics Committee of Yijishan Hospital, The First Affiliated Hospital of Wannan Medical College, and all guardians of the children included in the study signed an informed consent form indicating voluntary consent to participating in the study.

### Statistical analysis

Statistical analyses were conducted via SPSS 26.0 software (IBM, Armonk New York, USA) and R software (version 4.3.1). Categorical variables are reported as counts and percentages and were evaluated via chi-square tests or Fisher's exact tests, as appropriate. Continuous variables are reported as the mean ± standard deviation (SD) and were evaluated via a *t*-test or the Kruskal–Wallis test, as appropriate. All variables were explored by univariate analysis, and variables with *P* < 0.1 in univariate analysis were entered into the multivariate logistic regression analysis. The data are expressed as ORs and 95% confidence intervals (CIs). *P* < 0.05 was considered statistically significant.

## Results

### Baseline patient characteristics

After excluding participants aged ≥18 years or <6 years, those with incomplete data and those who were unwilling to complete the questionnaire survey, we included a total of 1,187 children with valid questionnaires with an age range of 6–17 years from three public hospital examination centers in Wuhu. Among the included children, 621 were male (52.3%) and 566 were female (47.7%).

### Incidence rates of RAP and *Helicobacter pylori* infection in children

According to the diagnostic criteria of RAP, among the 1,187 participants, 182 were diagnosed with RAP (15.3%), including 86 males (47.3%) and 96 females (52.7%). The results of ^13^C-UBT revealed that among the 1,187 children, 266 were infected with *Helicobacter pylori*, with an infection rate of 22.4%, including 137 males (51.5%) and 129 females (48.5%).

### Risk factors for RAP in children

The subjects were divided into two groups: the RAP group and the non-RAP group. Multivariate regression analysis revealed that academic stress (OR = 1.378, 95% CI: 1.009–1.713) and less than 1 h physical activity per day (OR = 1.616, 95% CI: 1.528–1.863) were independent risk factors for RAP. RAP was not associated with sex, BMI, frequent of fast-food consumption, or *Helicobacter pylori* infection status. The study subjects were divided into two age groups: 6–11 years and 12–17 years. Although the incidence of RAP was greater in younger children than in older children, logistic regression analysis revealed that age was not an independent risk factor for RAP (OR = 1.294, 95% CI: 0.930–1.801) (Table 1).

**Table 1:** Univariate and multivariate analyses of demographic characteristics and *Helicobacter pylori* infection in the RAP and non-RAP groups.

Characteristics	RAP (*n* = 182)	Non-RAP (*n* = 1,005)	*P* ^1^	Multivariate analyses		
				OR	95% CI	*P* ^2^
Sex			0.613			
Male	86 (47.3%)	535 (53.2%)				
Female	96 (52.7)	470 (46.8%)				
Age			0.002			
6–11 years	127 (69.8%)	568 (56.5%)		1.294	0.930–1.801	0.126
12–17 years	55 (30.2%)	437 (43.5%)		1.00		
BMI	21.2 ± 3.9	21.3 ± 4.1	0.137			
*Helicobacter pylori* infection						
*H. Pylori* positive	37 (20.3%)	229 (22.8%)	0.459			
*H. Pylori* negative	145 (79.7%)	776 (77.2%)				
Study pressure			<0.001			
Yes	114 (62.6%)	465 (46.3%)		1.378	1.009–1.713	0.009
No	68 (37.4%)	540 (53.7%)				
Daily physical activity time			<0.001			
<1 h	120 (65.9%)	483 (48.1%)		1.616	1.528–1.863	<0.001
≥1 h	62 (34.1%)	522 (51.9%)				
Fast-food			0.269			
Occasionally	36 (19.8%)	242 (24.1%)				
Sometimes	89 (48.9%)	526 (52.4%)				
Often	57 (31.3%)	236 (23.5%)				

The values are expressed as the means ± SDs or *n* (%), *P*^1^ values from the univariate analyses, and *P*^2^ values from the multivariate analyses.

### Risk factors for *Helicobacter pylori* infection in children

An investigation of the *Helicobacter pylori*-positive group and *Helicobacter pylori*-negative group revealed that age (OR = 2.139, 95% CI: 1.757–4.219), left-behind status (OR = 1.813, 95% CI: 1.318–2.981), lack of handwashing before meals (OR = 2.257, 95% CI: 1.821–4.471), low maternal education level (OR = 2.629, 95% CI: 1.725–4.163), paternal history of *Helicobacter pylori* infection (OR = 1.923, 95% CI: 1.226–3.018), maternal history of *Helicobacter pylori* infection (OR = 4.153, 95% CI: 2.735–6.306), washing dishes in a basin (OR = 3.250, 95% CI: 1.921–5.498), and not disinfecting tableware (OR = 2.146, 95% CI: 1.447–3.182) were independent risk factors for *Helicobacter pylori* infection in children in Wuhu, China. *Helicobacter pylori* infection was not related to sex, place of residence (urban or rural), frequency of tooth brushing, or total household income. High paternal education level was a protective factor against *Helicobacter pylori* infection (OR = 0.538, 95% CI: 0.357–0.811) (Table 2).

**Table 2:** Univariate and multivariate analyses of the demographic characteristics of the participants in *H. Pylori*-positive and *H. Pylori*-negative groups.

Characteristic	*H. Pylori* positive	*H. Pylori* negative		Multivariate	Analyses	
	(*n* = 266)	(*n* = 921)	*P* ^1^	OR	95% CI	*P* ^2^
Sex			0.983			
Male	137 (51.5%)	484 (52.6%)				
Female	129 (48.5%)	437 (47.4%)				
Age			<0.001			
6–11 years	77 (28.9%)	618 (67.1%)		1.00		
12–17 years	189 (71.1%)	303 (32.9%)		2.139	1.757–4.219	<0.001
Resident location			0.363			
Urban	197 (74.1%)	713 (77.4%)				
Rural	69 (25.9%)	208 (22.6%)				
Left-behind children			0.001			
Yes	168 (63.2%)	449 (48.8%)		1.813	1.318–2.981	<0.001
No	98 (36.8%)	472 (51.2%)		1.00		
Brushing frequency			0.862			
≤1	146 (54.9%)	528 (57.3%)				
≥2	120 (45.1%)	393 (42.7%)				
Habits of handwashing before meals			<0.001			
No	157 (59.0%)	366 (39.7%)		2.257	1.821–4.474	<0.001
Yes	109 (41.0%)	555 (60.3%)		1.00		
Education level of father			0.024			
College and above	92 (34.6%)	397 (43.1%)		0.538	0.357–0.811	0.003
Middle/high school	174 (65.4%)	524 (56.9%)		1.00		
Education level of mother			<0.001			
College and above	108 (40.6%)	542 (58.8%)		1.00		
Middle/high school	158 (59.4%)	379 (41.2%)		2.629	1.725–4.163	<0.001
Father's history of *Helicobacter pylori* infection			<0.001			
Positive	85 (32.0%)	174 (18.9%)		1.923	1.226–3.018	0.004
Negative	181 (68.0%)	747 (81.1%)		1.00		
Mother ’s history of *Helicobacter pylori* infection			<0.001			
Positive	118 (44.4%)	137 (14.9%)		4.153	2.735–6.306	<0.001
Negative	148 (55.6%)	784 (85.1%)		1.00		
Dishwashing			0.002			
In a basin	70 (26.3%)	136 (14.8%)		3.250	1.921–5.498	<0.001
Tap water	196 (73.7%)	785 (85.2%)		1.00		
Tableware disinfection			<0.001			
No disinfection	182 (68.4%)	414 (45.0%)		2.146	1.447–3.182	<0.001
Regular disinfection	84 (31.6%)	507 (55.0%)		1.00		
Annual household income (thousand yuan)			0.254			
<100	142 (53.4%)	465 (50.5%)				
≥100	124 (46.6%)	456 (49.5%)				

The values are expressed as the means ± SDs or *n* (%), *P*^1^ values from the univariate analyses, and *P*^2^ values from the multivariate analyses.

## Discussion

The aim of this study was to investigate the relationship between RAP and *Helicobacter pylori* infection in children in Wuhu, China as well as the risk factors for *Helicobacter pylori* infection in this region. We randomly selected 1,187 children from the physical examination centers of three public hospitals in Wuhu, China. Our survey revealed that the incidence rate of RAP in children was 15.3%. The *Helicobacter pylori* infection rate in the RAP group was 20.3%, and the *Helicobacter pylori* infection rate in the non-RAP group was 22.8%. There was no significant difference between the two groups. We think that RAP may not be associated with *Helicobacter pylori* infection. Korotkaya Y et al. (19) reported that although *Helicobacter pylori* infection is prevalent in low- and middle-income countries, most children have no symptoms and that there is no association between *Helicobacter pylori* infection and RAP. Wauters L et al. (20) found that RAP in children is associated with increased eosinophils in the duodenum and not with *Helicobacter pylori* infection. These studies are essentially in agreement with our survey results.

However, there are also different opinions, suggesting that *Helicobacter pylori* infection can induce RAP in children. Alimohammadi H et al. (21) conducted a survey of 1,558 school-aged children (aged 6–13 years) in Iran and reported that the prevalence of RAP was 9.3%. The *Helicobacter pylori* infection rate in children with RAP was significantly greater than that in healthy controls, indicating that *Helicobacter pylori* infection is an important risk factor for RAP in children. A survey in Romania (22) on *Helicobacter pylori*-positive children revealed that RAP was the most common symptom in children infected with *Helicobacter pylori*. Habib HS et al. (23) conducted a cross-sectional survey of high school students in Saudi Arabia and reported that, compared with *Helicobacter pylori*-negative students, *Helicobacter pylori*-positive students were significantly correlated with symptoms such as recurrent abdominal pain, anorexia, and nausea. The relationship between RAP and *Helicobacter pylori* infection in children remains controversial, and large-scale multicenter studies are needed to provide more evidence in the future.

Currently, the risk factors for RAP in children remain unclear. Our research revealed that sex, age, and fast-food consumption are not associated with RAP, whereas <1 h/day physical activity and academic stress are associated with RAP in children. Some studies have reported that dietary factors, mental stress, abnormal brain-gut axis interactions, eosinophilic gastroenteritis, and other factors may be involved in the pathogenesis of RAP in children (11–13). In China, most parents are accustomed to forcing their children to attend an excessive number of tutoring classes. Children spend more time studying but less time engage in physical activities, which can easily lead to anxiety and depression, resulting in physical discomfort and the occurrence of functional disorders.

Although our investigation found no association between *Helicobacter pylori* infection and RAP in children, *Helicobacter pylori* infection remains one of the common pathogenic bacteria that poses a great threat to human health. Recent studies have confirmed the increasingly common pathological role of *Helicobacter pylori* even in asymptomatic children (24, 25). *Helicobacter pylori* infection can lead to delayed growth and development in children (26).

*Helicobacter pylori* infection is distributed globally. Our research found that the *Helicobacter pylori* infection rate in children in Wuhu area was 22.4%. A meta-analysis of Li Xingchuan et al. (27) on *Helicobacter pylori* infection in Chinese children revealed that the overall *Helicobacter pylori* infection rate in the Chinese natural population of children and adolescents was 29%, with significant regional distribution differences. The *Helicobacter pylori* infection rate in areas with a high incidence of gastric cancer was 2.8 times higher than that in areas with a low incidence of gastric cancer. The *Helicobacter pylori* infection rate in children in Wuhu area was lower than that reported by Li Xingchuan, indicating it may be related to the decline in the *Helicobacter pylori* infection rate in China in recent years, and Wuhu is a non-gastric cancer epidemic area.

A meta-analysis involving 198 articles and 152,650 children (28) revealed that the overall prevalence of *Helicobacter pylori* infection in children worldwide is 32.3%. The prevalence of *Helicobacter pylori* infection in low- and middle-income countries is significantly greater than that in high-income countries (29). Due to differences in race, regional environment, lifestyle habits, demographic characteristics, sample size, and testing methods among research subjects, the survey results of *Helicobacter pylori* infection in children worldwide also differ, with infection rates ranging between 30.5% and 40.1% (30).

Furthermore, we divided the research subjects into two age groups and found that the *Helicobacter pylori* infection rate in children aged 12–17 was higher than that in children aged 6–11. Ren S et al. (31) reported that China is a country with a high incidence of *Helicobacter pylori* infection, ranging from 28.0% in children and adolescents to 46.1% in adults, and the risk of *Helicobacter pylori* infection increases with age, which is essentially consistent with our research findings.

Hygiene habits are closely associated with *Helicobacter pylori* infection. Our investigation revealed that having poor hygiene habits, such as not washing hands before meals, using a basin to wash dishes, and not disinfecting tableware, and having a family history of *Helicobacter pylori* infection are risk factors for *Helicobacter pylori* infection in children. Studies have shown that (26, 27) more siblings or children, sharing rooms, lack of a sewage treatment system, and history of maternal *Helicobacter pylor*i infection are risk factors for *Helicobacter pylori* infection in children, which is consistent with the our findings. The relationship between brushing frequency and *Helicobacter pylor*i infection remains controversial. Research has reported (32) that brushing teeth ≤1 per day is a risk factor for *Helicobacter pylori* infection. However, our study revealed that *Helicobacter pylori* infection in children is not associated with Brushing frequency.

The relationship between parental education level and *Helicobacter pylori* infection in children has garnered considerable attention in recent years. Our investigation shows that low maternal education level is an independent risk factor for *Helicobacter pylori* infection. Polivanova TV et al. (33) reported that in children in the Caucasus region of Russia, there is a negative correlation between *Helicobacter pylori* infection rates and maternal education levels. This is consistent with our research. Additionally, our survey indicates that a father's high level of education is a protective factor in preventing *Helicobacter pylori* contamination.

It is generally held that place of residence and household income may be associated with *Helicobacter pylori* infection. In the current study, we found that *Helicobacter pylori* infection in children in Wuhu area is neither related to whether they reside in rural or urban areas, nor to their family's economic income. Previous research reported (34) that living in rural areas and low total household income are risk factors for *Helicobacter pylori* infection. This is inconsistent with our research findings and might be attributed to the developed economy, rural urbanization, and reduced urban‒rural disparities in Wuhu area in recent years.

Additionally, our research revealed that being left behind is an independent risk factor for *Helicobacter pylori* infection in children in Wuhu area. Chinese left-behind children are generally taken care of by elderly family members over 60 years old. In developing countries, the elderly population has a low education level and poor hygiene habits (35), which may be the main reason for *Helicobacter pylori* infection in left-behind children.

The primary strength of our study is that it was a multicenter study on the relationship between RAP and *Helicobacter pylori* infection in children in Wuhu, China, and the risk factors for *Helicobacter pylori* infection in children in this region with a large sample size. This study provides epidemiological evidence for the prevention and treatment of RAP and *Helicobacter pylori* infection. There are also limitations to this study. Our survey subjects were limited to the Wuhu area, and surveys were not conducted in other regions of China. Our sample is not representative of all Chinese children. Moreover, regional differences may also result in our research findings not reflecting the actual situation of China's overall child and youth population. Therefore, it is necessary to conduct larger-scale surveys or comparative studies across multiple regions in the future.

## Conclusions

The incidence rate of RAP in children in Wuhu, China, is 15.3%. RAP in children was not associated with *Helicobacter pylori* infection, but strongly associated with <1 h/day physical activity and academic stress. It is necessary to pay attention not only to the clinical symptoms but also to the physical activity and academic stress of the children. Only by combining physical and mental treatment can the optimal treatment be provided for children with RAP. In addition, our investigation revealed that the *Helicobacter pylori* infection rate among children in Wuhu area was 22.4%. Left-behind status, poor hygiene habits, low maternal education level, and family history of *Helicobacter pylori* infection are risk factors for *Helicobacter pylori* infection in children. Our study may provide epidemiological evidence for the prevention and treatment of *Helicobacter pylori* infection in this region.

## Data Availability

The datasets presented in this article are not readily available because due to the confidentiality agreement of this study, the original data cannot be provided. If there is any doubt about the data, we will try our best to provide more detailed explanations. Requests to access the datasets should be directed to Yong Gu, guyongnn@126.com.

## References

[1] Reust CE Williams A. Recurrent abdominal pain in children. Am Fam Physician. (2018) 97(12):785–93. 30216016

[2] Andrews ET Beattie RM Tighe MP. Functional abdominal pain: what clinicians need to know. Arch Dis Child. (2020) 105(10):938–44. 10.1136/archdischild-2020-318825 32152039

[3] Ayonrinde OT Ayonrinde OA Adams LA Sanfilippo FM O' Sullivan TA Robinson M The relationship between abdominal pain and emotional wellbeing in children and adolescents in the raine study. Sci Rep. (2020) 10(1):1646. 10.1038/s41598-020-58543-0 32015372 PMC6997389

[4] Bradshaw S Brinkley A Scanlan B Hopper L. The burden and impact of recurrent abdominal pain—exploring the understanding and perception of children and their parents. Health Psychol Behav Med. (2022) 10(1):888–912. 10.1080/21642850.2022.2121710 36186891 PMC9518242

[5] Holstein BE Damsgaard MT Ammitzbøll J Madsen KR Pedersen TP Rasmussen M. Recurrent abdominal pain among adolescents: trends and social inequality 1991–2018. Scand J Pain. (2021) 21(1):95–102. 10.1515/sjpain-2020-0062 32892190

[6] Calvano C Warschburger P. Treatment for pediatric functional abdominal pain: an initial examination of reciprocal associations between pain, functional impairment, and parental distress. J Pediatr Psychol. (2022) 47(4):483–96. 10.1093/jpepsy/jsac011 35237811

[7] Owiredua C Flink I Boersma K. Prevalence and risk factors for pain-specific school absenteeism in adolescents with recurrent pain: a prospective population-based design. Eur J Pain. (2023) 27(3):390–400. 10.1002/ejp.2065 36478020

[8] Ragnarsson S Johansson K Bergström E Sjöberg G Hurtig AK Petersen S. Recurrent pain in school-aged children: a longitudinal study focusing on the relation to academic achievement. Pain. (2022) 163(11):2245–53. 10.1097/j.pain.0000000000002625 35250010

[9] Ragnarsson S Myleus A Hurtig AK Sjöberg G Rosvall P Petersen S. Recurrent pain and academic achievement in school-aged children: a systematic review. J Sch Nurs. (2020) 36(1):61–78. 10.1177/1059840519828057 30786840

[10] Myint K Jacobs K Myint AM Lam SK Lim YA Boey CC Psychological stresses in children trigger cytokine- and kynurenine metabolite-mediated abdominal pain and proinflammatory changes. Front Immunol. (2021) 12:702301. 10.3389/fimmu.2021.702301 34539633 PMC8442661

[11] Quek SH. Recurrent abdominal pain in children: a clinical approach. Singapore Med J. (2015) 56(3):125–8; quiz 132. 10.11622/smedj.2015038 25820843 PMC4371190

[12] Bremner AR Sandhu BK. Recurrent abdominal pain in childhood: the functional element. Indian Pediatr. (2009) 46(5):375–9. 19478350

[13] Piriyakitphaiboon V Sirinam S Noipayak P Sirivichayakul C Pornrattanarungsri S Limkittikul K. Risk factors for recurrent abdominal pain in children with nonorganic acute abdominal pain. Pediatr Gastroenterol Hepatol Nutr. (2022) 25(2):129–37. 10.5223/pghn.2022.25.2.129 35360380 PMC8958051

[14] Jones NL Koletzko S Goodman K Bontems P Cadranel S Casswall T Joint ESPGHAN/NASPGHAN guidelines for the management of *Helicobacter pylori* in children and adolescents (update 2016). J Pediatr Gastroenterol Nutr. (2017) 64(6):991–1003. 10.1097/MPG.0000000000001594 28541262

[15] Shahinyan T Amaryan G Tadevosyan A Braegger C. Clinical, endoscopic, and histological characteristics of *Helicobacter pylori* positive and negative Armenian children with recurrent abdominal pain and/or dyspepsia. Georgian Med News. (2022) 324:71–8. 35417865

[16] Yu X Feng D Wang G Dong Z Zhou Q Zhang Y. Correlation analysis of *Helicobacter pylori* infection and digestive tract symptoms in children and related factors of infection. Iran J Public Health. (2020) 49(10):1912–20. 10.18502/ijph.v49i10.4694 33346225 PMC7719659

[17] Nguyen J Kotilea K Bontems P Miendje Deyi VY. *Helicobacter pylori* infections in children. Antibiotics (Basel). (2023) 12(9):1–16. 10.3390/antibiotics12091440 37760736 PMC10525885

[18] Chobot A Porębska J Krzywicka A Żabka A Bąk-Drabik K Pieniążek W No association between *Helicobacter pylori* infection and gastrointestinal complaints in a large cohort of symptomatic children. Acta Paediatr. (2019) 108(8):1535–40. 10.1111/apa.14721 30656740

[19] Korotkaya Y Shores D. *Helicobacter pylori* in pediatric patients. Pediatr Rev. (2020) 41(11):585–92. 10.1542/pir.2019-0048 33139411

[20] Wauters L Harris PR Walker MM Serrano CA Villagrán A Rakhra GS Letter: childhood recurrent abdominal pain is associated with increased duodenal eosinophilia independent of *Helicobacter pylori* infection. Aliment Pharmacol Ther. (2023) 58(1):134–6. 10.1111/apt.17556 37307550

[21] Alimohammadi H Fouladi N Salehzadeh F Alipour SA Javadi MS. Childhood recurrent abdominal pain and *Helicobacter pylori* infection, Islamic Republic of Iran. East Mediterr Health J. (2017) 22(12):860–4. 10.26719/2016.22.12.860 28181660

[22] Rosu OM Gimiga N Stefanescu G Anton C Paduraru G Tataranu E *Helicobacter pylori* infection in a pediatric population from Romania: risk factors, clinical and endoscopic features and treatment compliance. J Clin Med. (2022) 11(9):1–13. 10.3390/jcm11092432 35566557 PMC9099726

[23] Habib HS Hegazi MA Murad HA Amir EM Halawa TF El-Deek BS. Unique features and risk factors of *Helicobacter pylori* infection at the main children’s intermediate school in Rabigh, Saudi Arabia. Indian J Gastroenterol. (2014) 33(4):375–82. 10.1007/s12664-014-0463-1 24777895

[24] Manfredi M Gismondi P Iuliano S. Is *Helicobacter pylori* anyway pathogen in children? Inquiry. (2023) 60:469580231154650. 10.1177/00469580231154650 36803205 PMC9940224

[25] Emerenini FC Nwolisa EC Iregbu FU Eke CB Ikefuna AN. Prevalence and risk factors for *Helicobacter pylori* infection among children in Owerri, Nigeria. Niger J Clin Pract. (2021) 24(8):1188–93. 10.4103/njcp.njcp_687_20 34397029

[26] Erturk EY Karaman U Arici YK Top S Yolalan G. Factors influencing *Helicobacter pylori* positivity in children. Niger J Clin Pract. (2021) 24(5):685–91. 10.4103/njcp.njcp_595_19 34018978

[27] Xingchuan L Haidong W Ni Z Yuping W Yongning Z. Systematic review and meta-analysis of epidemiological investigation of *Helicobacter pylori* infection in children and adolescents in China. J Clin Pediatr. (2017) 35(10):782–7. (Article is in Chinese).

[28] Yuan C Adeloye D Luk TT Huang L He Y Xu Y The global prevalence of and factors associated with *Helicobacter pylori* infection in children: a systematic review and meta-analysis. Lancet Child Adolesc Health. (2022) 6(3):185–94. 10.1016/S2352-4642(21)00400-4 35085494

[29] Okuda M Lin Y Kikuchi S. *Helicobacter pylori* infection in children and adolescents. Adv Exp Med Biol. (2019) 1149:107–20. 10.1007/5584_2019_361 31037557

[30] Chen YC Malfertheiner P Yu HT Kuo CL Chang YY Meng FT Global prevalence of *Helicobacter pylori* infection and incidence of gastric cancer between 1980 and 2022. Gastroenterology. (2024) 166(4):605–19. 10.1053/j.gastro.2023.12.022 38176660

[31] Ren S Cai P Liu Y Wang T Zhang Y Li Q Prevalence of *Helicobacter pylori* infection in China: a systematic review and meta-analysis. J Gastroenterol Hepatol. (2022) 37(3):464–70. 10.1111/jgh.15751 34862656

[32] Liébana J García-Casas V Liébana-Cabanillas F Arias-Moliz MT. Prevalence of the colonization of *Helicobacter pylori* among students of the school of dentistry, University of Granada, Spain. Med Oral Patol Oral Cir Bucal. (2016) 21(5):e573–8. 10.4317/medoral.21167 27475692 PMC5005094

[33] Polivanova TV Malaty H Vshivkov VA. Epidemiology *Helicobacter pylori* infection in children in the Tyva Republic (Russia). Helicobacter. (2022) 27(3):e12882. 10.1111/hel.12882 35285106

[34] Lim SH Kwon JW Kim N Kim GH Kang JM Park MJ Prevalence and risk factors of *Helicobacter pylori* infection in Korea: nationwide multicenter study over 13 years. BMC Gastroenterol. (2013) 13:104. 10.1186/1471-230X-13-104 23800201 PMC3702482

[35] Schacher K Spotts H Correia C Walelign S Tesfaye M Desta K Individual and household correlates of *Helicobacter pylori* infection among young Ethiopian children in Ziway, Central Ethiopia. BMC Infect Dis. (2020) 20(1):310. 10.1186/s12879-020-05043-1 32334539 PMC7183626

